# Festival to Commemorate the Introduction of the Potato

**Published:** 1845-05

**Authors:** 


*Festival to Commemorate the introduction of the Potato.*—Several of the German States have, we learn from the *Atheneum*, instituted feasts in honor of the introduction of the potato; and the anniversary of its importation has just been held as a jubilee at Bavaria. At Menterschwaige, near Munich, a festival was observed on the occasion in which dishes of the poor man's especial root, variously dressed, had the place of honor on the table:—while the bust of Sir Francis Drake, crowned with garlands of oak, and presented to the commune, for the occasion, by its sculptor Schwanthaler, occupied the centre of the hall. In France, a monument is about to be erected to Parmentier, commemorating its introduction into that country. It may appear to our readers, that the honor paid to the memory of Drake was really due to Raleigh; but it is probable that the Germans are literally correct. The first colonists, sent out by Raleigh, were disheartened when Drake touched at Virginia, and he consented to bring them home. Lane, the governor, who is believed to have brought with him the first tobacco, may, and probably did, bring the first potato; if so, though indebted to the enterprise of Raleigh for the discovery, it was Drake's ship that actually introduced the first root.

